# Impact of Preanalytical and Analytical Methods on Cell-Free DNA Diagnostics

**DOI:** 10.3389/fcell.2021.686149

**Published:** 2021-09-06

**Authors:** Jure Krasic, Irena Abramovic, Alen Vrtaric, Nora Nikolac Gabaj, Sasa Kralik-Oguic, Ana Katusic Bojanac, Davor Jezek, Nino Sincic

**Affiliations:** ^1^Department of Medical Biology, School of Medicine, University of Zagreb, Zagreb, Croatia; ^2^Scientific Group for Research on Epigenetic Biomarkers, School of Medicine, University of Zagreb, Zagreb, Croatia; ^3^Centre of Excellence for Reproductive and Regenerative Medicine, School of Medicine, University of Zagreb, Zagreb, Croatia; ^4^Department of Clinical Chemistry, Sestre Milosrdnice University Hospital Center, Zagreb, Croatia; ^5^Faculty of Pharmacy and Biochemistry, University of Zagreb, Zagreb, Croatia; ^6^Clinical Institute of Laboratory Diagnostics, University Hospital Centre Zagreb, Zagreb, Croatia; ^7^Department of Histology and Embryology, School of Medicine, University of Zagreb, Zagreb, Croatia

**Keywords:** cell-free DNA, liquid biopsy, blood plasma, seminal plasma, preanalytics, cell-free DNA methylation, cell-free DNA integrity

## Abstract

While tissue biopsy has for the longest time been the gold-standard in biomedicine, precision/personalized medicine is making the shift toward liquid biopsies. Cell-free DNA (cfDNA) based genetic and epigenetic biomarkers reflect the molecular status of its tissue-of-origin allowing for early and non-invasive diagnostics of different pathologies. However, selection of preanalytical procedures (including cfDNA isolation) as well as analytical methods are known to impact the downstream results. Calls for greater standardization are made continuously, yet comprehensive assessments of the impact on diagnostic parameters are lacking. This study aims to evaluate the preanalytic and analytic factors that influence cfDNA diagnostic parameters in blood and semen. Text mining analysis has been performed to assess cfDNA research trends, and identify studies on isolation methods, preanalytical and analytical impact. Seminal and blood plasma were tested as liquid biopsy sources. Traditional methods of cfDNA isolation, commercial kits (CKs), and an in-house developed protocol were tested, as well as the impact of dithiothreitol (DTT) on cfDNA isolation performance. Fluorimetry, qPCR, digital droplet PCR (ddPCR), and bioanalyzer were compared as cfDNA quantification methods. Fragment analysis was performed by qPCR and bioanalyzer while the downstream application (cfDNA methylation) was analyzed by pyrosequencing. In contrast to blood, semen as a liquid biopsy source has only recently begun to be reported as a liquid biopsy source, with almost half of all publications on it being review articles. Experimental data revealed that cfDNA isolation protocols give a wide range of cfDNA yields, both from blood and seminal plasma. The addition of DTT to CKs has improved yields in seminal plasma and had a neutral/negative impact in blood plasma. Capillary electrophoresis and fluorometry reported much higher yields than PCR methods. While cfDNA yield and integrity were highly impacted, cfDNA methylation was not affected by isolation methodology or DTT. In conclusion, NucleoSnap was recognized as the kit with the best overall performance. DTT improved CK yields in seminal plasma. The in-house developed protocol has shown near-kit isolation performance. ddPCR LINE-1 assay for absolute detection of minute amounts of cfDNA was established and allowed for quantification of samples inhibited in qPCR. cfDNA methylation was recognized as a stable biomarker unimpacted by cfDNA isolation method. Finally, semen was found to be an abundant source of cfDNA offering potential research opportunities and benefits for cfDNA based biomarkers development related to male reproductive health.

## Graphical Abstract

Depiction of the experimental design.

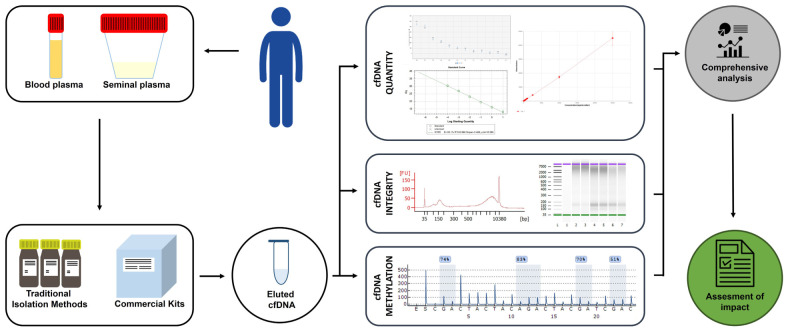

## Introduction

Tissue biopsies have been the gold-standard in disease diagnostics and prognostics (Constâncio et al., 2020). However, the issues with tissue biopsies have become more apparent, such as its invasiveness, the need for repetitive sampling, the problem of tumor heterogeneity, and unapproachability of certain tissues (Stewart et al., 2018; Constâncio et al., 2020; Lee E. Y. et al., 2020). With the advent of precision and personalized medicine these constraints are becoming both more evident and limiting. Utilization of the genetic material in liquid biopsies offers solutions in a reliable, cost-effective, and minimally invasive way (Lobo et al., 2019; Constâncio et al., 2020; Spiller et al., 2020; Thakral et al., 2020; Wang et al., 2020; Vandekerkhove et al., 2021).

Cell-free DNA (cfDNA) is one of the most prominent molecules present in liquid biopsies, discovered in 1948 by Mandel and Metai in human blood plasma (Aucamp et al., 2016; Cook et al., 2018; Krasic et al., 2018). Originating from apoptosis, necrosis, and direct cell secretion, cfDNA has been detected in almost all body fluids, such as urine, sputum, cerebrospinal fluid, pleural fluid, cyst fluid, saliva, bronchial lavages, and semen (Draškovič, 2017; Chen et al., 2018; Kumar et al., 2018; Johansson et al., 2019). It reflects the genetic and epigenetic characteristics of its tissue of origin, such as DNA methylation, mutations, or microsatellite alterations (Barták et al., 2019). cfDNA is double-stranded and varies in size, ranging from 180 bp up to 10,000 bp, with the majority being around 180 bp long, corresponding to the size of the DNA within the nucleosomes by apoptotic processes (Draškovič, 2017; Cook et al., 2018; Somen et al., 2019; Streleckiene et al., 2019; Li S. et al., 2020). In the blood of healthy individuals cfDNA quantity ranges from 1.8 to 44 ng/ml (Draškovič, 2017; Streleckiene et al., 2019), mostly originating from hematopoietic cells (Aucamp et al., 2016; Somen et al., 2019; Lampignano et al., 2020). Its quantity and fragment size can be increased by conditions such as physical activity, pregnancy, and various other states (Warton and Samimi, 2015; Cook et al., 2018; Barták et al., 2019; Johansson et al., 2019; Ottaviano et al., 2021). Disease-derived cfDNA primarily originates from necrotic and phagocytotic processes, such as in inflammation, sepsis, trauma, or cancer, and is elevated in quantity (Aucamp et al., 2016; Ponti et al., 2019; Somen et al., 2019; Yu et al., 2019; Li S. et al., 2020; Ottaviano et al., 2021). Among these cfDNA fractions, circulating tumor DNA (ctDNA), which originates from cancer cells, is of great interest as a novel oncological biomarker (Solassol et al., 2018; Keup et al., 2020; Ponti et al., 2020).

Biomarkers associated with different diseases have been successfully detected in cfDNA, such as copy number variations, single nucleotide polymorphisms, cfDNA integrity, and epigenetic modifications including cfDNA methylation (Vaissière et al., 2009; Vaca-Paniagua et al., 2015; Trigg et al., 2018; Lee et al., 2019; Constâncio et al., 2020; Li S. et al., 2020; Rodriguez-Casanova et al., 2021; Yin et al., 2021). In particular cfDNA quantity, integrity, and methylation have shown themselves to be the most prominent diagnostic parameters (Trigg et al., 2018; Mbaye et al., 2019; Lampignano et al., 2020; Lo et al., 2021; Vandekerkhove et al., 2021; Zhang et al., 2021). cfDNA methylation presents itself as a valuable biomarker in liquid biopsy research due to its high specificity for various biological states and robust biological stability (Constâncio et al., 2020; Miller et al., 2021), with some authors even presenting cfDNA methylation status as the universal cancer biomarker (Meddeb et al., 2019). Recent reports highlight multiple possible applications of the cfDNA methylation profiling among which are: being able to identify the tissue of origin and discern between different tissues (healthy and cancerous) (Moss et al., 2018; Stewart et al., 2018; Gaga et al., 2020; Sprang et al., 2020; Miller et al., 2021), as well as guiding therapy selection and monitoring disease prognosis (Somen et al., 2019; Tuaeva et al., 2019; Li S. et al., 2020). For example, LINE-1 cfDNA hypomethylation is associated with multiple cancer characteristics, such as risk, type, progression, and poor prognosis (Lee et al., 2019). Further research has shown a correlation between cfDNA quantity and tumor size, metastasis status, and burden (Stewart et al., 2018; Streleckiene et al., 2019). The cfDNA integrity index (CFI) is yet another biomarker for cancer diagnostics and therapy monitoring (Kumar et al., 2018; Chan et al., 2021) with longer fragments described in cancer patients (Meddeb et al., 2019; Shi et al., 2020; Chan et al., 2021). Research on the possible applications of cfDNA’s size profile is ongoing in many fields such as oncology, transplantation medicine, cardiology, and infectious disease medicine (Shi et al., 2020), as well as in reproductive medicine since sperm fragmentation index has been proposed to supplement routine diagnostics in identifying subfertility issues and predict *in vitro* fertilization success (Li L. et al., 2020). Limitations of cfDNA include the relatively short half-life and the variable amount of target cfDNA fraction (Ponti et al., 2019; Sorber et al., 2019; Lampignano et al., 2020). In particular, ctDNA is detected in blood samples of over 75% pancreatic, ovarian, and colorectal cancer patients, while in prostate cancer patients significantly lower levels were found (Ponti et al., 2019) suggesting that for different pathologies different liquids should be investigated as biopsies.

In contrast to blood plasma, very little research has been done on seminal plasma despite many factors pointing to its great promise in male reproductive medicine. For example, seminal plasma has a comparatively high amount of cfDNA of heterogeneous size in relation to other body fluids (Draškovič, 2017; Ponti et al., 2019) which allows for analysis such as the genome-wide promoter methylation of the human testis and epididymis to be performed (Wu et al., 2013). Seminal plasma cfDNA levels correlate with sperm parameters, with the quantity of low molecular weight cfDNA correlating to positive sperm parameters, and higher cfDNA concentrations discriminating azoospermic patients from normozoospermic (Li et al., 2009; Chen et al., 2018; Ponti et al., 2018; Mbaye et al., 2019; Morgan and Watkins, 2020). So far, cfDNA concentrations and their electrophoretic patterns have been shown to discriminate between prostate cancer, benign prostatic hyperplasia patients, and age-matched healthy controls (Ponti et al., 2019). This could allow seminal plasma to provide a non-invasive alternative to prostate tissue biopsies which carry risks of morbidity (Drabovich et al., 2014; Ponti et al., 2018). Seminal plasma is a true non-invasive biopsy, and with the high abundance of cfDNA is suitable for both cost-effective and more advanced diagnostic methods, promising to find a place in male reproductive medicine (Li et al., 2009; Drabovich et al., 2014; Ponti et al., 2018, 2019; Mbaye et al., 2019; Morgan and Watkins, 2020).

Although progress has been made many issues have remained unsolved, leaving the integration of cfDNA into routine clinical practice facing the challenge of standardization of both the preanalytical phase and analytical assays (Lampignano et al., 2020). Currently, there are over 60 clinical trials involving cfDNA approaches, which all depend on their practical advantage and robustness in the clinical setting (Kumar et al., 2018). So far, cfDNA is only being utilized by specialized laboratories which have reported 13 different techniques for the isolation of cfDNA, 5 different methodological approaches for quantification, and 11 different genotyping methods, clearly demonstrating little inter-laboratory standardization (Haselmann et al., 2018; Kumar et al., 2018; Lampignano et al., 2020). Sample collection, preservation, blood collection tube type, storage temperature, serum or plasma preparation, and cfDNA extraction were already proven to have an impact on the amount, integrity, and purity of isolated cfDNA with a strong influence on the downstream molecular analysis (Medina Diaz et al., 2016; Sorber et al., 2019; Lee J. S. et al., 2020; Samoila et al., 2020). A number of studies are being published on assessing various preanalytical variables and technical aspects of cfDNA extraction which are calling attention to new potential issues in the employed methodology and a need for greater standardization in liquid biopsy protocols and workflows (Page et al., 2006; Malentacchi et al., 2015; Draškovič, 2017; Cook et al., 2018; Kumar et al., 2018; Mojtabanezhad Shariatpanahi et al., 2018; Barták et al., 2019; de Kock et al., 2019; Meddeb et al., 2019; Sorber et al., 2019; Streleckiene et al., 2019; Abramovic et al., 2020; Augustus et al., 2020; Bronkhorst et al., 2020; Lampignano et al., 2020; Lee E. Y. et al., 2020; Lee J. S. et al., 2020; Salvianti et al., 2020; Samoila et al., 2020). The clinical use of cfDNA also requires the development and standardization of high sensitivity methods able to analyze highly fragmented DNA (Johansson et al., 2019; Sorber et al., 2019). Despite all this, comprehensive studies examining the impact of extraction methods on cfDNA diagnostic parameters are sorely lacking in both blood and seminal plasma (Draškovič, 2017). With both the preanalytical protocols and analytical assays standardized, reference values correlating to disease states could be reliably identified and translated to clinical practice (Aucamp et al., 2017).

The aim of this study was to identify and evaluate these preanalytic and analytic factors that influence cfDNA diagnostic parameters in blood and semen samples. For this reason, the impact of traditional cfDNA isolation methods and kit-based isolation methods was evaluated on cfDNA diagnostic parameters (cfDNA yield, integrity, and cfDNA methylation). We have employed fluorimetry, qPCR, digital droplet PCR (ddPCR), and capillary electrophoresis as the four most commonly used cfDNA detection methods. CFI was assessed using qPCR and capillary electrophoresis. The impact on cfDNA methylation was analyzed by pyrosequencing.

## Materials and Methods

### Text Mining

In order to identify all studies comparing cfDNA isolation methods and cfDNA research in general, a Europe PMC based literature search was conducted, with the last update being on the 31st of December 2020. Europe PMC was selected due to it being a flexible platform with currently 1.3 billion annotations text mined from articles, and growing (Ferguson et al., 2021). Keywords were combined with Boolean operators into search terms and are listed in Table 1.

**TABLE 1:** List of search terms used in the literature search and the goal for which they were used.

Research goal	Search term
Identification of publications on cfDNA isolation methods	(“cell-free DNA” OR “cell free DNA” OR cfDNA) AND (isolation method OR extraction method OR purification method) AND (optimization OR comparison OR selection OR evaluation) AND (plasma OR serum OR blood OR urine OR ejaculate OR semen OR “seminal fluid” OR “liquid biopsy”)
Identification of publications on cfDNA research	“cell-free DNA” OR “cfDNA” OR “cell free DNA”
	(“cell-free DNA” OR “cfDNA” OR “cell free DNA”) AND (blood)
	(“cell-free DNA” OR “cfDNA” OR “cell free DNA”) AND (urine)
	(“cell-free DNA” OR “cfDNA” OR “cell free DNA”) AND (semen OR ejaculate OR “seminal plasma” OR “seminal fluid”)

### Sample Collection and Plasma Preparation

Blood and ejaculate samples were taken from 12 normozoospermic men. The criteria for normozoospermia were according to the guidelines of World Health Organization (WHO) (Cooper et al., 2009). Samples were drawn and plasma was prepared at the clinical hospital centers Zagreb and Sestre milosrdnice.

Of peripheral venous blood 12 ml (two 6 ml tubes) was collected into EDTA-containing tubes (Greiner Bio-One) and processed within 2 h after venipuncture. To ensure cell-free plasma collection and to prevent cellular contamination, all EDTA-blood samples were centrifuged in two steps (1400 × *g* for 10 min and then 4500 × *g* for 10 min). Blood plasma was stored at −80°C.

Ejaculate samples were obtained by masturbation after 3–5 days of sexual abstinence and were allowed to liquefy for 30–60 min at room temperature. Seminal plasma was obtained by triple centrifugation of ejaculate samples to prevent cellular contamination (400 × *g* for 10 min, 12,000 × *g* for 10 min, and 20,000 × *g* for 10 min), which is a modification of the protocol by Li et al. (2009). Seminal plasma was stored at −80°C.

Plasma samples were pooled before further processing into one blood plasma sample and one seminal plasma sample, to remove subject variance.

### cfDNA Isolation

The most commonly used traditional methods for cfDNA isolation (TIM) were selected for the study: the Triton–Heat–Phenol (THP) by Xue et al. (2009), Phenol–chloroform isoamyl alcohol isolation (PCI) protocols by Yuan et al. (2012); Schmidt et al. (2005), and Hufnagl et al. (2013), which were all done following the author’s recommendations. We have also tested the salting-out method by Miller et al. (1988) which is one of the most widely used gDNA isolation methods and which has been previously used, with slight modifications in cfDNA isolation (Jorgez et al., 2006). The recommendations laid out in the original article were followed.

An in-house PCI cfDNA isolation protocol with dithiothreitol (DTT) was developed, following DTT amount recommendations for sperm gDNA isolation from the research article by Doerksen et al. (2000). Briefly, 100 μL of lysis solution (240 mM Tris–HCl pH 8.0, 1800 mM NaCl, and 120 mM EDTA), 120 μL of 10% SDS, 30 μL of proteinase-K, and 12 μL of 1 M DTT was added to 1 ml of blood plasma and seminal plasma, respectively. The plasma sample was vortexed, spun down, and left over-night in a heating block at 50°C. PCI was added 1:1 to the plasma, shaken, and incubated at room temperature for 5 min, after which the sample was centrifuged at 16,000 × *g* for 15 min. The supernatant was transferred to a new tube and the DNA was precipitated by adding 1/20 of the volume of 4 M NaCl and 1/1 volume of ice-cold ethanol and incubating at −20°C over-night. The DNA was centrifuged at maximum speed for 30 min, washed with 70% ethanol, centrifuged at maximum speed for 30 min and dissolved in 100 μL of TE buffer (10 mM Tris–HCl pH 8.0 and 1 mM EDTA).

The three most widely used commercial kits (CK) for cfDNA isolation were selected, Qiagen’s QIAmp Circulating Nucleic Acid Kit which is currently the gold standard (Streleckiene et al., 2019), Zymo’s Quick-cfDNA Serum & Plasma Kit, and Macherey-Nagel’s NucleoSnap cfDNA. All protocols were performed according to the manufacturer’s instruction, using the QIAvac 24 Plus vacuum station (Qiagen). The proteinase-K digestion step has been increased to overnight in all three kits, as it has been shown that longer digestion results in higher yields (Pérez-Barrios et al., 2016). Protocols used are depicted in Table 2.

**TABLE 2:** List of cfDNA isolation methods used in the study along with their sources.

cfDNA isolation methods used				
Protocol	Protocol description	Source	Previously tested on blood plasma	Previously tested on seminal plasma
TIM-1	Triton–Heat–Phenol (THP)	Xue et al., 2009	Yes	No
TIM-2	PCI	Yuan et al., 2012	Yes	No
TIM-3	PCI	Hufnagl et al., 2013	Yes	No
TIM-4	PCI	Schmidt et al., 2005	Yes	No
TIM-5	Salting out	Miller et al., 1988	Yes	No
TIM-6	PCI	In-house	No	No
TIM-7	PCI – TIM-3 modified	Hufnagl et al., 2013	No	No
CK-1	QIAmp Circulating Nucleic Acid Kit	Qiagen	Yes	Yes
CK-2	Quick-cfDNA Serum & Plasma Kit	Zymo	Yes	No
CK-3	NucleoSnap cfDNA	Macherey-Nagel	Yes	No

The impact of DTT on cfDNA isolation was tested as well, with the protocols described in Table 3. To the CK groups, 10 mM DTT was added since it is the standard concentration used in most sperm DNA isolations. To the modified Hufnagl et al.’s (2013) 80 mM DTT was added to test if the reported higher concentrations of DTT improve isolation yields (He et al., 2017).

**TABLE 3:** List of methods used to assess the impact of DTT addition on blood and seminal plasma isolation.

DTT impact on cfDNA				
Protocol	Protocol description	DTT amount (mM)	Previously tested on blood plasma	Previously tested on seminal plasma
TIM-6	In-house developed PCI	10	No	No
TIM-7	Modification of Hufnaghl et al.	80	No	No
CK-1 +	QIAmp Circulating Nucleic Acid Kit	10	No	No
CK-2 +	Quick-cfDNA Serum & Plasma Kit	10	No	No
CK-3 +	NucleoSnap cfDNA	10	No	No

The cfDNA isolation protocols were performed starting with 1 ml of blood or seminal plasma which was eluted in 100 μL of elution buffer (for CK) or TE buffer (for TIM). cfDNA of the same body liquid and isolated by the same protocol was pooled for fragment analysis.

### Fluorometric dsDNA Assay

To perform fluorometric quantification of cfDNA quantity Quant-iT PicoGreen dsDNA Assay Kit (Thermofisher), was used, following the manufacturer’s instructions. Samples were excited at 480 nm and the fluorescence was measured at 520 nm using a TECAN Spark multimode reader. Lambda DNA was used to generate the standard curve. All samples and standards were run in triplicates.

### Real-Time PCR

As described in detail in Rago et al. (2007), human LINE-1 is a retrotransposon family member with over 100,000 elements interspersed throughout the human genome. As such, quantifying human LINE-1 offers a sensitive method for quantifying human cfDNA, therefore the concentration and integrity of total cfDNA were determined by qPCR with primers targeting the second open reading frame of the human LINE-1 element.

Primers amplifying an 82-bp and a 224-bp LINE-1 region were used (Takai et al., 2015). The shorter amplicon was used to quantify total cfDNA, while the longer amplicon was used to calculate the CFI, with the ratio of longer cfDNA fragments to the shorter ones (Rostami et al., 2020). The primers used are listed in Table 4.

**TABLE 4:** List of primers used along with their amplicon size.

Target	Description		Sequence	bp
LINE-1	Short sequence	F	5′-TCACTCAAAGCCGCTCAACTAC-3′	82
		R	5′-TCTGCCTTCATTTCGTTATGTACC-3′	
LINE-1	Long sequence	F	5′-TCTGCCTTCATTTCGTTATGTACC-3′	224
		R	5′-TCAGCACCACACCACACCTATTC-3′	
LINE-1	Pyrosequencing	F	5′-BIOTIN-TAGGGAGTGTTAGATAGTGG-3′	108
		R	5′-AACTCCCTAACCCCTTAC-3′	
		SEQ	5′-CAAATAAAACAATACCTC-3′	

qPCR reactions were carried out in triplicate, using 1 μL of isolated cfDNA, SsoAdvanced Universal SYBR Green Supermix (Bio-Rad Laboratories), and 250 nM forward and reverse primers. Real-Time PCR amplification was performed on the CFX96 Touch Real-Time PCR Detection System (Bio-Rad Laboratories) according to the protocol: pre-cycling heat activation of DNA polymerase at 98°C for 3 min followed by 40 cycles of denaturation at 98°C for 10 s, annealing/extension at 60°C for 30 s, followed by a melting point gradient from 65 to 95°C. The analysis was performed using CFX Maestro Software (Bio-Rad Laboratories). Absolute quantification of cfDNA in each sample was determined by a standard curve with serial dilutions of human genomic DNA (Qiagen).

### Droplet Digital PCR

To assess the viability of cfDNA concentration detection by ddPCR, the total 82 bp LINE-1 cfDNA fragments were quantified on the ddPCR system (Bio-Rad Laboratories), using the modified protocol from He K. et al. (2019).

Isolated cfDNA samples were diluted (with dilutions ranging from 3× to 1000×) to allow for accurate quantification of LINE-1 fragments and avoiding droplet oversaturation. Commercial human genomic DNA (Qiagen) was used to create a standard curve ranging from 3.125 to 100 pg/μL for accurate absolute cfDNA quantification. All samples and standards were *HaeIII*-digested and were ran in duplicates. The ddPCR reaction was set up using 1 μL of isolated cfDNA, QX200 ddPCR EvaGreen, Supermix (Bio-Rad Laboratories), and 100 nM forward and reverse primers (Table 4). Droplets were generated in the Bio-Rad QX200 droplet generator (Bio-Rad Laboratories) using the QX200 Droplet Generation Oil for EvaGreen. The droplets were transferred to a 96 well ddPCR plate (Bio-Rad Laboratories) and heat-sealed with a pierceable aluminum foil (Bio-Rad) in the PX1 PCR Plate Sealer (Bio-Rad). PCR amplification was done using the CFX96 Deep Well PCR thermal cycler (Bio-Rad Laboratories) according to the protocol: activation at 95°C for 5 min, 40 cycles of denaturation at 95°C for 30 s, and annealing/extension at 60°C for 60 s, followed by signal stabilization at 4°C for 5 min and 90°C for 5 min and the final step being an infinite hold at 4°C. All steps had a ramp rate of 2°C/s. Droplets were analyzed in the QX200 Droplet Reader (Bio-Rad Laboratories) using QuantaSoft software and quantified assuming Poisson’s random distribution. Data was acquired using one-dimensional or two-dimensional based plotting systems as recommended by the manufacturer. Thresholds were set by excluding only the true negative population, according to the no-template control which was included in each assay.

### Bioanalyzer

The isolated cfDNA fragment profile and concentration were analyzed using capillary electrophoresis. The High Sensitivity DNA microchip kit (Agilent Technologies) and an Agilent 2100 Bioanalyzer (Agilent Technologies) equipped with Expert 2100 software were used to perform the analysis, according to the instructions provided by the manufacturer. After the nucleic acids were separated analogously to capillary electrophoresis, they were normalized to the two DNA markers and were visualized as a virtual band. Fragments were separated into short (223 bp and less) and long (224 bp and more), which has allowed the calculation of CFI, with the ratio of longer fragments to total quantified cfDNA.

### cfDNA Methylation Analysis

Bisulfite conversion was performed using the EpiTect Plus DNA Bisulfite Kit (Qiagen) and 40 μL of the isolated cfDNA, according to the manufacturer’s instructions. Bisulfite-converted cfDNA was then eluted in 30 μL of elution buffer.

For the PCR amplification of the LINE-1 repetitive region using the PyroMark PCR Kit (Qiagen) 1 μL of bisulfite-treated DNA was used as the template. Samples along with methylated and unmethylated control DNA from the EpiTect PCR Control DNA kit (Qiagen) were run in triplicates. PCR protocol was as follows: initial denaturation at 95°C for 15 min; 50 cycles of denaturation at 94°C for 30 s, annealing at 58°C for 30 s, and extension at 72°C for 30 s; final extension was at 72°C for 10 min (Daskalos et al., 2009). The biotinylated PCR product was purified using the Pyromark Q24 Vacuum Workstation (Qiagen). Methylation levels of the six CpG’s were then measured by Pyromark Q24 Advanced System with PyroMark Q24 CpG Advanced Reagents (Qiagen). cfDNA methylation levels were calculated as the ratio of C/T at a CpG site using the Pyromark Q24 Advanced Software 3.0.1 (Qiagen). Global cfDNA methylation was calculated as the average value of the six CpG’s. Primers used in cfDNA methylation analysis are listed in Table 4.

### Statistical Analysis

Isolated cfDNA concentrations were statistically analyzed by the Kruskal–Wallis test with Dunn’s multiple correction test using the GraphPad Prism Software (GraphPad), with the level of significance set to 0.05.

## Results

### Literature Mining

**FIGURE 1:**
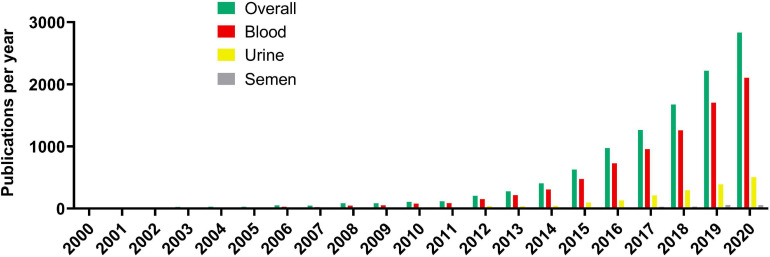
Cell-free DNA article publication rate per year.

The literature search using Europe PMC for scientific publications involving cfDNA research has found the earliest two publications in 1967 while reaching up to 10 publications per year only in 1988. This low-publishing trend on cfDNA research existed until the 2000s when more than 10 publications per year were published consistently. The following increase in publication quantity regarding cfDNA was extremely rapid, with the first 6 years of the 21st century producing more articles than the whole period from 1967 to 2000 (Figure 1). The increase is still ongoing with 100 publications per year being achieved in 2010, 1000 per year in 2017 while in 2020 there were 2833 publications related to cfDNA. However, of the published articles 2105 included blood as the cfDNA source, urine being a distant second with 504 published articles, and semen (seminal fluid) has lagged even further behind with only 55 published articles as a source of cfDNA.

**FIGURE 2:**
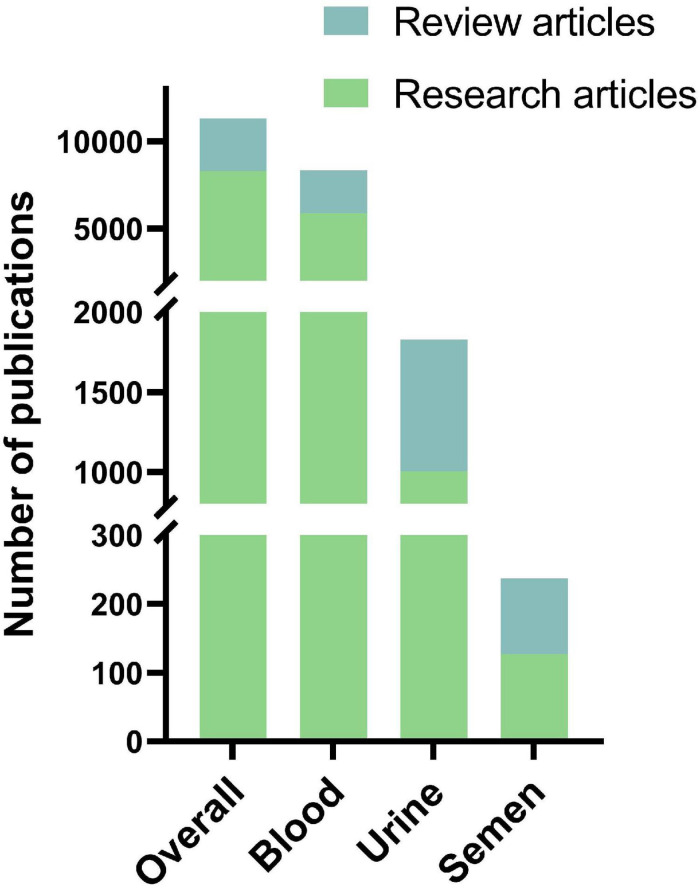
Amount of original research articles and review articles in the total number of publications on cfDNA within respective body fluid of origin.

Publications on cfDNA research were then separated by type, being original research or review articles (Figure 2). Overall, review articles make up 27% of all articles published on cfDNA, 29% of all articles on blood, 45% on urine, and 46% on semen as a cfDNA source.

**FIGURE 3:**
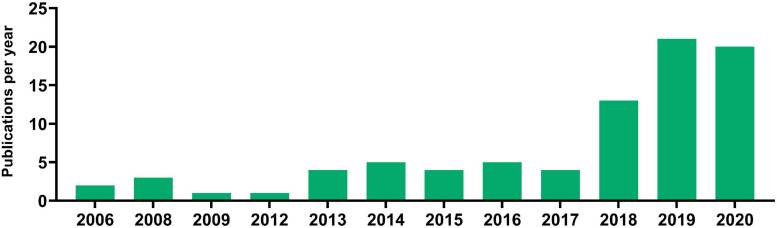
Articles published investigating the impact of various protocols on cfDNA research and analysis.

Next, Europe PMC literature search was carried out to identify publications that compare cfDNA isolation methods and the impact of various preanalytical factors on them. The search has produced 2198 results, of which 696 were discarded due to being reviews, thus leaving 1502 results. Poster and oral presentations were further excluded leaving 1393 results for review by title and abstract by two authors. Finally, 147 articles were left for full-text evaluation by two authors, who have additionally excluded articles with no comparison of different cfDNA isolation methods, with no investigation on the impact of preanalytical parameters on cfDNA isolation, and no usage of human samples. Eighty-three publications were obtained at the end, dating as early as 2006 with a frequency not more than a few publications per year, until 2018 where 13 articles were published. The increase in interest for cfDNA isolation methods continued in 2019 and 2020 with 21 and 20 research articles being published, respectively (Figure 3). The selected articles, when separated according to body fluid used for cfDNA isolation conform with the overall cfDNA article trends, blood as a liquid biopsy was generally the most explored, followed by urine and so far, only one published research was done on seminal plasma as cfDNA source dating from 2009 (Table 5). Most of the publications have compared the impact of different isolation methods using only one liquid biopsy source, followed by assessing the potential of body fluids (such as bronchial lavage or urine) for cfDNA isolation, while only rare studies compared the efficacy impact of different isolation methods on different body fluids.

**TABLE 5:** Investigated parameters within the publications assessing the impact of various preanalytical and analytical parameters of cfDNA research.

	Articles comparing cell-free DNA methods		83
cfDNA source	Plasma		65
	Blood		
	Serum		5
	Urine		15
	Semen		1
Comparisons		Method	58
	Isolation		
	Between liquids		9
	Centrifugation		9
	Quantification/fragment distribution		31
	Tubes/preservatives		13
	Effect of time and temperature		15
	Preanalytical impacts on analysis outcome		42

### cfDNA Yield

**FIGURE 4:**
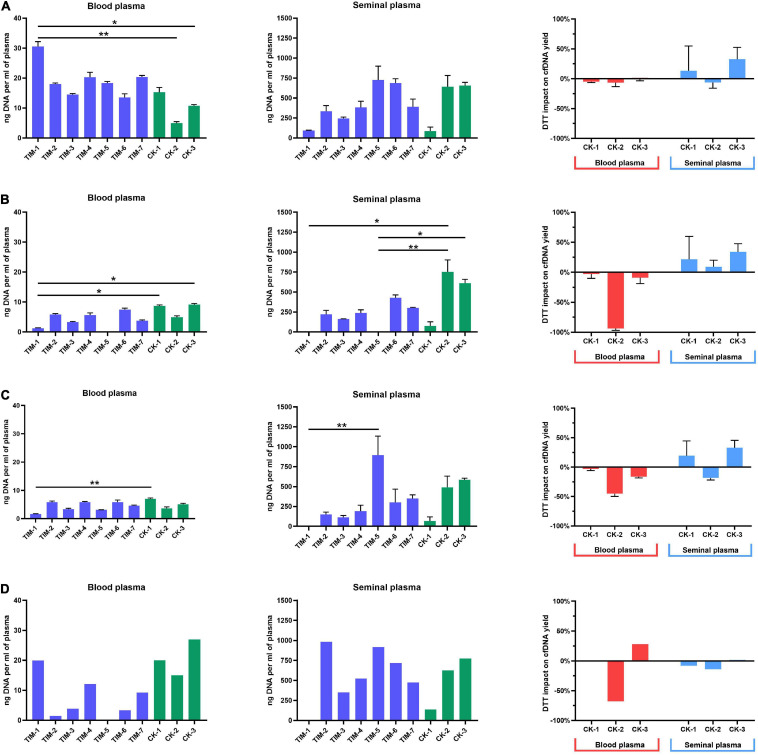
Cell-free DNA yields from blood and seminal plasma. **(A)** Yields measured by fluorometry, with DTT impact on commercial kits depicted as percentage change; **(B)** yields measured by qPCR analysis of LINE-1, with DTT impact on commercial kits depicted as percentage change; **(C)** yields measured by ddPCR, with DTT impact on commercial kits depicted as percentage change; **(D)** yields measured by capillary electrophoresis, with DTT impact on commercial kits depicted as percentage of change. Values represented are means with SD. Asterisks were used to depict statistical significance, *P < 0.05, **P < 0.01.

Fluorometric assessment of cfDNA yield has shown a significant impact of isolation methods on cfDNA yield in both blood and seminal plasma. Yields of cfDNA extracted from blood plasma according to TIM protocols were in the range from 14 to 31 ng/ml, while CK protocols have produced a narrower yield range from 5 to 15 ng/ml. Seminal plasma cfDNA yields from TIM protocols have ranged from 95 to 727 ng/ml, while CK protocols have ranged from 86 to 656 ng/ml (Figure 4A). As seen in blood plasma, most of the TIM’s outperformed the CK’s, while in seminal plasma TIM-5, TIM-6, CK-2, and CK-3 samples stood out roughly equally above the rest. Moreover, in blood plasma, the addition of DTT in three CK has led to a reduction of 5, 6, and 1% of cfDNA yield, respectively. In seminal plasma, an increase in yield by 14% in CK-1, and by 33% in CK-3 was observed, while in CK-2 a reduction of 6% was found.

Assessment of cfDNA yield by qPCR analysis of LINE-1 has again shown a significant impact of different isolation methods on blood and seminal plasma cfDNA yield (Figure 4B). However, contrary to the fluorometric measurement, in blood plasma, cfDNA yields from TIM protocols measured by qPCR were lower (1–7 ng/ml) than those from CK samples (5–9 ng/ml). A similar trend was observed for seminal plasma, with higher (75–753 ng/ml) yields of CK samples compared to TIM (0.2–429 ng/ml). Furthermore, we observed a negative impact of DTT addition to CK protocols on cfDNA yields in blood plasma with a decrease of 3% in CK-1, 94% in CK-2, and 9% in CK-3. In seminal plasma, however, an improvement of cfDNA yields was obtained by the addition of DTT with 22% in CK-1, 9% in CK-2, and 34% in CK-3. However, both the blood and seminal plasma cfDNA samples isolated by the TIM-5 protocol failed to be detected by the qPCR measurement.

Quantification of cfDNA yield by ddPCR analysis of LINE-1 has shown the same overall trend established by qPCR analysis (Figure 4C). In the blood plasma processed by TIM protocols, cfDNA yields have ranged from 1 to 6 ng/ml, while in CK protocols a range of 4–7 ng/ml, was obtained. In the seminal plasma sample processed by TIM cfDNA yields have ranged from 1 to 895 ng/ml with TIM-5 having had the highest yield overall, while in CK protocols the range was a bit smaller, from 68 to 584 ng/ml. With ddPCR employed as a measurement tool, the addition of DTT to blood plasma in CK protocols has reduced cfDNA yields by 4% in CK-1, 43% in CK-2, and 14% in CK-3. The seminal plasma sample has shown an increase in cfDNA quantity by 22% when processed by CK-1, and 28% by CK-3, while a reduction of 19% was found in CK-2 protocol after the addition of DDT.

Capillary electrophoretic analysis of cfDNA quantity from blood plasma has shown similar yields to fluorometric analysis, with TIM protocol ranges having been in the range of 1–20 ng/ml and CK yields were in the range of 15–27 ng/ml. The highest yield was exhibited by CM-3 protocol at 26 ng/ml (Figure 4D). The TIM-5 protocol, however, was not analyzable, similarly to qPCR analysis. In seminal plasma, the same overall trend was observed, with yields again being more similar to fluorometric analysis. cfDNA yields from TIM protocols were in the range from 350 to 982 ng/ml with TIM-2 having had the highest yield at 982 ng/ml. Meanwhile, the TIM-1 protocol was not analyzable (as in both the qPCR and ddPCR analysis). As for cfDNA yields from CK protocols, they were in the range of 138–775 ng/ml. The addition of DTT to blood plasma has reduced yield in the CK-2 protocol by 67%, while it has increased the yield of the CK-3 protocol by 28%. As for the impact of DTT on the cfDNA yield of blood plasma processed by CK-1, it was not analyzable by the bioanalyzer. In seminal plasma, DDT has reduced overall yield by 8% in CK-1, by 14% in CK-2, and by 1% in CK-3.

### cfDNA Integrity

**FIGURE 5:**
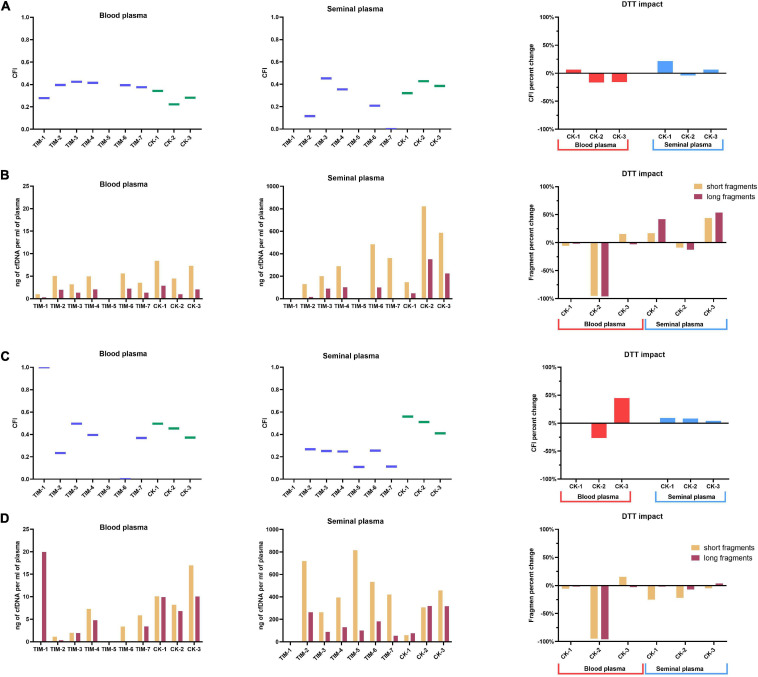
Cell-free DNA fragmentation index. **(A)** CFI analysis by qPCR, with the impact of DDT on long fragment ratio; **(B)** total long and short LINE-1 cfDNA fragments quantified by qPCR, with the impact of DTT on total fragment yield; **(C)** CFI analysis by capillary electrophoresis, with the impact of DTT on long fragment ratio; **(D)** total long and short LINE-1 cfDNA fragments quantified by capillary electrophoresis, with the impact of DTT on total fragment yield. Values represented are means.

Analysis of the ratio of short (82 bp) and long (224 bp) LINE-1 fragments by qPCR has allowed us to quantify the total amount of long and short LINE-1 fragments in samples, and subsequently calculate the CFI for the cfDNA isolation methods (Figure 5A). Blood plasma CFI has ranged from 0.22 to 0.43, while seminal plasma CFI has ranged from 0 to 0.45. Most blood plasma protocols have exhibited a similar CFI of 0.4, with the lowest CFI having been exhibited by the CK-2 protocol, which has presented a lower amount of isolated fragments in general (Figure 5B). Seminal plasma CFI has shown a much higher variability depending on the isolation method, with only TIM-3, TIM-4, and the CK protocols having had comparable levels of CFI. It is also noteworthy that the seminal plasma processed by TIM-7 only contained short cfDNA fragments. The addition of DTT to blood plasma increased the CFI by 6% in CK-1 but reduced it in CK-2 and CK-3 by 16%. In seminal plasma, CK-1 has produced an increase of the CFI by 22% and CK-3 by 6%, while CK-2 saw a reduction of 4% due to DDT.

Analysis of fragment size was performed by capillary electrophoresis as well, with the cut-off set at 223 bp to discriminate between long and short cfDNA fragments. In blood plasma, CFI has ranged from 0.2 to 1.0, along with the fact that only long fragments have been detected in the TIM-1 protocol (Figure 5C). In seminal plasma, the CFI has ranged from 0.1 up to 0.6, while cfDNA isolated by TIM-1 has not been able to be analyzed. The largest amount of long cfDNA fractions was isolated using TIM-2, CK-2, and CK-3 protocols in seminal plasma (Figure 5D). The impact of DTT addition on the CFI of blood plasma in CK protocols was not analyzable in CK-1, while a 26% reduction in CK-2 and an increase of 44% in CK-3 was observed. In seminal plasma, the addition of DTT has increased CFI in CK-1 by 9%, CK-2 by 8%, and CK-3 by 4%.

The addition of DTT to CK-3 protocol in blood plasma sample has led to increased fragmentation of isolated cfDNA, with an increase in short fragments and decrease in long ones, while DTT has usually produced either a uniform increase or decrease in both long and short fragment amount.

### cfDNA Methylation

**FIGURE 6:**
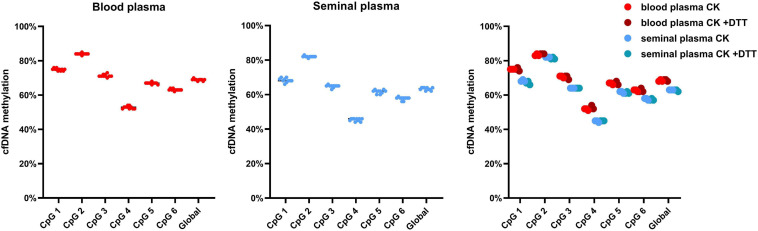
Cell-free DNA methylation of LINE-1 in blood and seminal plasma. cfDNA methylation is depicted both as CpG specific and global, along with the impact of DTT on cfDNA methylation. Values represented are means. Every dot represents a different protocol used.

We have assessed both the global and CpG specific cfDNA methylation of LINE-1 isolated from blood and seminal plasma by pyrosequencing. Here, no impact of either cfDNA isolation methods or the addition of DTT on cfDNA methylation levels has been found (Figure 6). Additionally, in seminal plasma global cfDNA hypomethylation of 6% in comparison to blood plasma was detected, with the detected blood plasma cfDNA methylation level of 69% and seminal plasma level of 63%. CpG 2 had the smallest difference between blood and seminal plasma cfDNA methylation, with blood plasma having had 84% methylation compared to 82% in seminal plasma. While CpG 4 had the largest difference, with blood plasma having had 53% cfDNA methylation compared to 45% in seminal plasma.

## Discussion

As the literature analysis has shown, interest in cfDNA research is only increasing year to year, from 10 publications per year in the 1990s to over 2800 publications in 2020. However, this rise in popularity hasn’t been followed by increased standardization in methods for isolation, quantification, and analysis of cfDNA, all of which lead to potential pitfalls when comparing results obtained from different methodologies (Trigg et al., 2018). This is accentuated by the fact that in the last 3 years more than half of all publications investigating the impact of different isolation methods or parameters impacting cfDNA yield were published. However, there is no clear consensus on the methodology using blood plasma as the body fluid of origin for cfDNA research, and so far, only one study has investigated this in seminal plasma, meaning this is far from a closed topic. With male reproductive system disorders affecting the quality of men’s lives both in their prime and in the aging population, their treatment is in part limited by the prejudice related to check up’s and the absence of accurate diagnostic methods (Drabovich et al., 2014). Seminal plasma promises to complement medical imaging and blood-based tests for non-invasive diagnostics of male reproductive health, with male infertility, prostate, and testicular cancer being pathologies most likely to see the greatest benefits (Drabovich et al., 2014).

With regards to plasma preparation, centrifugation protocols widely differ but previous research has shown the least amount of cellular DNA contamination when a two-step centrifugation protocol is used, with no negative impact on cfDNA yield (Pös et al., 2020). A two-step centrifugation protocol is the most common in blood plasma preparation, consisting of a slow speed first step (<2000 × *g*) followed by a high-speed second step (>3000 × *g*) (Sorber et al., 2019; Pös et al., 2020). While this is sufficient for blood plasma preparation and is according to the WHO guidelines for seminal plasma preparation (Cao et al., 2011; Pös et al., 2020) we have had multiple issues with the two-step approach in seminal plasma preparation for cfDNA analysis. Despite other studies successfully using this method for seminal plasma preparation (Li et al., 2009; Ponti et al., 2019; Eikmans et al., 2020; Fraczek et al., 2020) we have found that seminal plasma processed by standard double centrifugation still has too much cellular debris present. This debris can often result in spin column blockage at worst and at best increase the time required for the sample to pass through the column by a significant amount. In this research, a three-step method with an additional centrifugation step at 20,000 × *g* has removed most of the cellular debris allowing for unimpeded cfDNA isolation.

It has been demonstrated that preanalytical parameters, including cfDNA isolation methods, impact diagnostic parameters, notably the yield, quality, degree of cfDNA fragmentation, and cfDNA methylation (Barták et al., 2019; Johansson et al., 2019; Oreskovic et al., 2019). However, despite the research using cfDNA rapidly increasing very little is known on the exact degree of impact these parameters have on the diagnostic parameters and especially in semen. Furthermore, the results of cfDNA analysis vary according to the method employed (Bronkhorst et al., 2020). To investigate the impact of the selected preanalytical and analytical methods on the downstream cfDNA diagnostic parameters we have evaluated in this work the most commonly used methods of cfDNA isolation. We have analyzed both traditional methods and those based on CKs, as well as routine methods for cfDNA quantification: a fluorometric dsDNA assay, qPCR, and ddPCR assays and capillary electrophoresis (Ponti et al., 2018; Streleckiene et al., 2019). We were able to assess for potential discrepancies, downstream inhibitors, and confounding factors.

We have confirmed the previously reported higher yields of cfDNA from seminal plasma (Ponomaryova et al., 2020) regardless of the isolation method. Higher yields allow for easier overall sample processing of both high and low sensitivity downstream analytical methods allowing for greater application in male reproductive medicine, especially oncology and fertility management (Ponti et al., 2019). We have also noticed a discrepancy between PCR and fluorometric quantification of cfDNA yield both in blood and seminal plasma isolated samples, with fluorometry suggesting greater quantities for all TIM’s. Fluorometric quantification of cfDNA has been reported to overestimate cfDNA quantity possibly due to cfDNA fragmentation, standards used (Sedlackova et al., 2013), or even due to the presence of carrier-RNA during isolation such as in CK-1 (Streleckiene et al., 2019). Quantification by qPCR has shown a reduction in the obtained cfDNA yields, with the exception of TIM-1 and TIM-5 which seem to have inhibited the qPCR reaction altogether. In CK-2 and CK-3 protocols, the cfDNA yields detected correspond the most between those obtained by PCR methods and fluorimetry and also show the least variability irrespective of the detection method used. We have also reported the successful application of a modified LINE-1 ddPCR assay for cfDNA quantification from liquid biopsy samples, which has allowed for absolute quantification of cfDNA. The quantification by ddPCR was more in line with yields obtained by qPCR, which seem to suggest the fluorimetric overestimation, but has also allowed for detection of samples which were inhibited in the qPCR analysis, most notably samples TIM-1 and TIM-5. This could be of importance for highly valuable, low-volume samples or those with the presence of inhibitors, giving ddPCR a supplementary role to qPCR cfDNA quantification, which is currently the gold standard (Streleckiene et al., 2019; Yan et al., 2021). Quantification of cfDNA samples by capillary electrophoresis has shown yields more similar to the ones obtained by fluorometric analysis. However, in seminal plasma cfDNA obtained by TIM-1 and TIM-5 as well as the blood plasma cfDNA obtained by CK-1 protocol, there could be the presence of possible contaminants or inhibitory substances in the buffer which prevent the detection of molecular markers and subsequent analyses. Protocols TIM-4, TIM-6, and TIM-7, as well as CK-3, have produced the highest cfDNA yields with the highest purity in both blood and seminal plasma samples with TIM-6 and CK-3 being our recommendation.

To our knowledge, no research has been done yet on the effect of DTT on cfDNA isolation, despite the fact that DTT has been used for the purpose of increasing DNA yield in extraction protocols due to its ability to reduce disulfide bonds and keep proteins in a reduced state (Leite et al., 2019). Due to this, DTT has found a wide application in isolation of DNA from sources like bacteria, highly mucous liquids, and semen (Doerksen et al., 2000; Radford et al., 2015; Liu et al., 2018), in concentrations commonly ranging from 1 to 10 mM DTT (Doerksen et al., 2000; Pacheco et al., 2011; Radford et al., 2015). However, concentrations of DTT up to 200 mM have also been tested for the effect on DNA yield (He et al., 2017; Liu et al., 2018) where optimal DNA yields were obtained by higher (160 and 200 mM) DTT concentrations. In our research, among TIM protocols we have designed two protocols, TIM-6 and TIM-7, that included DTT in concentrations of 10 and 80 mM, respectively. While the addition of DTT has resulted in increased cfDNA yields in both seminal and blood plasma there was no further increase with higher concentrations. However, in seminal plasma cfDNA there was even a reduction in CFI with a higher concentration of DTT. This can be explained by the well-known effect of DTT, which causes single-stranded nicks in double-stranded DNA (Fjelstrup et al., 2017). The observed increase in cfDNA yield has prompted us to modify all three investigated CK protocols with the addition of 10 mM DTT. However, what we have found is that while having a negligible or even a slightly negative effect on blood plasma cfDNA yield, it has greatly increased seminal plasma cfDNA yield. The addition of DTT has also impacted the CFI of obtained cfDNA, and while in cfDNA from blood plasma there is a noticeable increase in short versus long fragments, in seminal plasma cfDNA there is also an overall increase in both short and long fragments isolated. To summarize, this effect of DTT on cfDNA isolation could be of interest when working with small liquid biopsy volumes and low amounts of cfDNA and should be investigated further.

Overall, we presented four different methods with all showing the same general trend each with its strength and weakness (Bronkhorst et al., 2020). While certain isolation methods seem more prone to inhibitions in PCR-based analysis, they might still be analyzable using fluorometry, while capillary electrophoresis can be used for a technical overestimation check-up. Conversely, PCR methods can be applied to get a more accurate estimation of usable cfDNA fragments for downstream analyses, rather than just very short (less than 40 bp) fragments, such as seen in TIM processed seminal plasma cfDNA. In addition, the previously reported greater efficacy of CKs (Mauger et al., 2015; He Z. et al., 2019) was confirmed only in seminal plasma and not in blood plasma. Finally, our results show that the comparison of cfDNA yields obtained by different isolation and quantification methods is not feasible or reliable. While PCR methods offer greater consistency there is a place for non-PCR detection methods in supplementing PCR-based methods in cfDNA analysis, especially in the case of samples processed by traditional isolation methods (Keshavarz et al., 2015; Akbariqomi et al., 2019) which can contain inhibitory substances. Concerning the variability of cfDNA yield with regard to the isolation method used, blood plasma samples show the least method-dependent variability and especially when using PCR detection methods. However, statistically significant differences were detected between different isolation protocols in both blood and semen as cfDNA source, and by different methods of cfDNA quantification. While mitigation of the analytical bias can be done by employing multiple methods (Lampignano et al., 2020), the isolation method-dependent bias can only be solved by greater inter-lab standardization, especially when using liquid biopsies other than blood.

Optimal isolation protocols should extract all cfDNA fractions present in the sample, and not have a preference for a certain size (Johansson et al., 2019). In the case of blood plasma, we observed that TIM’s have a higher ratio of long cfDNA fragments, while CK’s have a larger quantity of short fragments. Seminal plasma in general has exhibited a distinct electrophoretic profile, with both small and large fragments being present, while in blood plasma the 180 bp cfDNA fragment has made up the majority of the cfDNA. This distinct profile has been proposed to be of diagnostic and clinical relevance (Ponti et al., 2019), meaning parameters impacting CFI must be paid attention to when working with semen as the source of liquid biopsy, especially if the analysis should be comparable to those done by other laboratories and centers. Capillary electrophoresis of blood plasma samples seems unable to detect the presence of larger fragments analyzable by qPCR, while certain samples unable to be amplified by PCR are able to be detected by this method. Complementary usage of both methods is required for fuller characterization of investigated samples. Evaluation of the impact of isolation methods on CFI has revealed that the impact is higher when working with semen than with blood plasma, also that the PCR detection methods produces more uniform results. This could be due to seminal plasma possessing a higher abundance of longer fragments of cfDNA, so it is also more susceptible to procedure induced fragmentation. Thus, when working with semen, attention should be brought to both isolation and detection methods employed, to avoid inducing fragmentation and analytical bias into a diagnostic parameter.

While studies have previously reported that isolation methods could impact cfDNA methylation analysis results (Barták et al., 2019), in this study we have found no impact. LINE-1 cfDNA methylation of the six investigated CpG’s as well as the average global methylation has been consistent across all analyzed isolation methods in both seminal and blood plasma. The consistency within individual CpG sites is especially important since averaging the methylation value of a CpG island often leads to a loss of relevant information (Sprang et al., 2020). Even in our case of 6% hypomethylation in seminal plasma samples without individual CpG analysis, there is no way of distinguishing if it is present in an individual site or across all sites. With pyrosequencing, we were able to reliably assess cfDNA methylation both globally and CpG site-specific even in samples with minute amounts of cfDNA and even in samples that contained PCR inhibitors. The requirement for eluted cfDNA to undergo bisulfite conversion and subsequent clean-up could have “leveled out” the different isolation protocols. This finding of the consistency of cfDNA methylation irrespective of isolation method opens new possibilities and opportunities in cfDNA biomarker research since it could bypass the whole issue of inter-lab standardization. cfDNA methylation could be reliably compared across labs and studies irrespective of preanalytical methods used.

**FIGURE 7:**
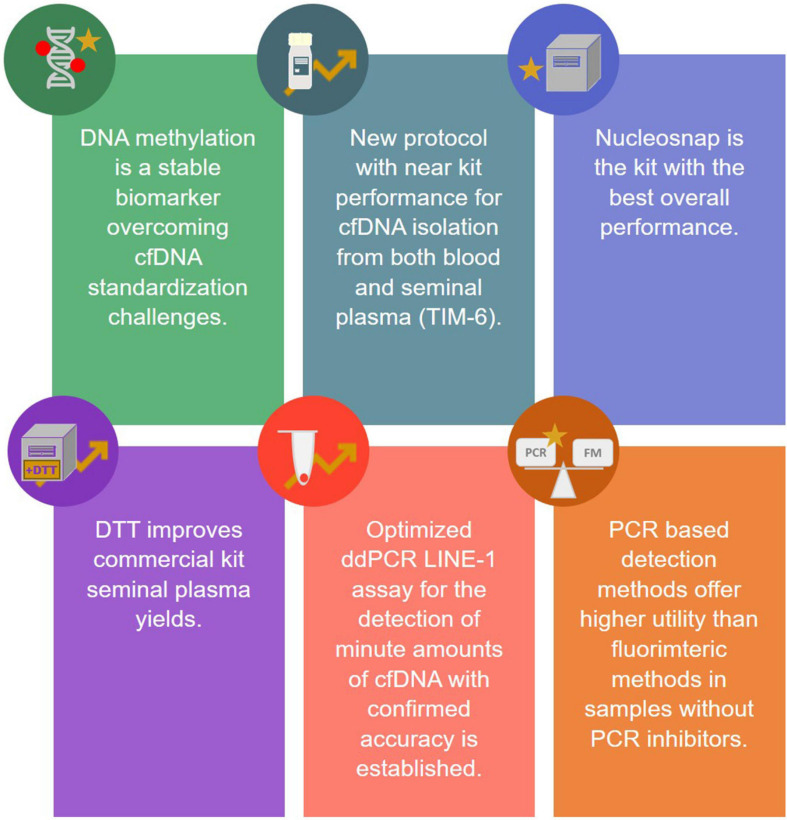
Key points. Short depiction of the takeaway points of this study.

To our knowledge, the current study is the first comprehensive investigation of preanalytical and analytical impact on diagnostic parameters of cfDNA obtained from blood and semen. According to the presented results, NucleoSnap is the best cfDNA isolation method in both blood and seminal plasma, overtaking the current *de facto* gold standard Qiagen. The in-house developed isolation protocol TIM-6 has presented itself as an inexpensive method but with near kit performance in both blood and seminal plasma cfDNA isolation (Figure 7). The addition of DTT could be beneficial especially when isolating cfDNA from seminal plasma. We suggest PCR based protocols for cfDNA detection due to their ease-of-use, and comparability of ddPCR and qPCR results. LINE-1 ddPCR assay is a reliable method for quantification of minute amounts of cfDNA applicable to hard-to-detect samples. Isolation methods have had no impact on cfDNA methylation results obtained by pyrosequencing. Presented results facilitate further cfDNA biomarker development, promoting standardization in related diagnostics and research. Finally, we stress that semen offers a promising source of cfDNA for male reproductive health research and patient management.

## Data Availability Statement

The raw data supporting the conclusions of this article will be made available by the authors, without undue reservation.

## Ethics Statement

The studies involving human participants were reviewed and approved by University of Zagreb School of Medicine (641-01/17-02/01). The patients/participants provided their written informed consent to participate in this study.

## Author Contributions

NS, JK, and AKB contributed to conception and design of the study. NS and DJ provided the funding and the resources required for the study. JK and IA performed the analysis. AV, NNG, and SK-O collected and processed the samples. NS, AKB, and DJ have administrated the project. NS, NNG, and SK-O have supervised the execution of the project. NS and JK have created the visuals for the manuscript. JK wrote the original draft of the manuscript. All authors contributed to manuscript revision, read, and approved the submitted version.

## Conflict of Interest

The authors declare that the research was conducted in the absence of any commercial or financial relationships that could be construed as a potential conflict of interest.

## Publisher’s Note

All claims expressed in this article are solely those of the authors and do not necessarily represent those of their affiliated organizations, or those of the publisher, the editors and the reviewers. Any product that may be evaluated in this article, or claim that may be made by its manufacturer, is not guaranteed or endorsed by the publisher.
